# Opioid tolerance in methadone maintenance treatment: comparison of methadone and levomethadone in long-term treatment

**DOI:** 10.1186/s12954-016-0095-0

**Published:** 2016-02-16

**Authors:** Stefan Gutwinski, Nikola Schoofs, Heiner Stuke, Thomas G. Riemer, Corinde E. Wiers, Felix Bermpohl

**Affiliations:** Department of Psychiatry and Psychotherapy, Charité Campus Mitte, Charité Universitätsmedizin Berlin, Charitéplatz 1, 10117 Berlin, Germany

**Keywords:** Opioid, Tolerance, Long-term, Maintenance treatment

## Abstract

**Background:**

This study aimed to investigate the development of opioid tolerance in patients receiving long-term methadone maintenance treatment (MMT).

**Methods:**

A region-wide cross-sectional study was performed focusing on dosage and duration of treatment. Differences between racemic methadone and levomethadone were examined. All 20 psychiatric hospitals and all 110 outpatient clinics in Berlin licensed to offer MMT were approached in order to reach patients under MMT fulfilling the DSM IV criteria of opiate dependence. In the study, 720 patients treated with racemic methadone or levomethadone gave information on the dosage of treatment. Out of these, 679 patients indicated the duration of MMT.

**Results:**

Treatment with racemic methadone was reported for 370 patients (54.5 %), with levomethadone for 309 patients (45.5 %). Mean duration of MMT was 7.5 years. We found a significant correlation between dosage and duration of treatment, both in a conjoint analysis for the two substances racemic methadone and levomethadone and for each substance separately. These effects remained significant when only patients receiving MMT for 1 year or longer were considered, indicating proceeding tolerance development in long-term treatment. When correlations were compared between racemic methadone and levomethadone, no significant difference was found.

**Conclusions:**

Our data show a tolerance development under long-term treatment with both racemic methadone and levomethadone. Tolerance development did not differ significantly between the two substances.

## Background

Methadone is currently the preferred drug of choice for the treatment of opioid dependence in many countries [1]. Methadone maintenance treatment (MMT) has been implemented to increase survival and stabilize patients, in order to enable them to reach opioid abstinence [2–4]. The advantages of MMT in opioid-dependent patients on mental and physical health and social integration have been described by reducing morbidity and mortality [1, 5–9], improving employment rates and reducing criminal activity [10–12].

Methadone is a synthetic opioid and was initially launched as an analgesic in 1939 [13]. It was introduced for the treatment of opioid dependence by Dole, Nyswander, and Kreek in 1964 [13, 14]. The long duration of methadone leads to a “narcotic blockade” and eliminates withdrawal symptoms for up to 36 h. Given in high doses, it reduces craving for heroin and blocks the effect of injected heroin, thereby freeing the patient from the daily cycle of seeking out, buying, and consuming heroin [1, 13–16].

Methadone has an asymmetric carbon atom resulting in two enantiomeric forms, the d- and l-isomers [17, 18]. The l-isomer has a 10 times higher affinity for the μ-opioid receptor than the d-isomer. Both racemic methadone (d-, l-methadone) and levomethadone (isolated l-methadone) are clinically used in all countries of the European Union and several countries worldwide [17, 19].

One potential problem of treatment with opioid agonists is the development of tolerance [20–22]. The effect of tolerance has been described for methadone during short treatment intervals of analgesia but seems to be reduced compared with other opioids, such as morphine, showing sometimes more than a 10-fold increase in dosage [18, 23]. It has been hypothesized that methadone’s ability to efficiently internalize the μ-opioid receptor and to exhibit an NMDA receptor antagonism may contribute to reduced opioid tolerance development compared with opioids such as morphine and oxycodone [18]. This antagonistic effect on the NMDA receptor is associated with both isomers of methadone. However, one could assume that the development of opioid tolerance is particularly attenuated under treatment with racemic methadone compared to levomethadone. That is, the d-enantiomer exhibits an antagonistic effect on NMDA receptors, but only a minimum opioid effect, resulting in a stronger NMDA-antagonistic effect per equivalence dose [24–27]. This would result in reduced tolerance development in racemic methadone compared to levomethadone.

The development of tolerance is of clinical relevance because it results in loss of efficacy of opioids and consequently in need of an increase of dosage [20–22]. To our knowledge, tolerance of methadone has not been examined over a long treatment period or in patients with opioid dependence in MMT. Additionally, differences of tolerance between racemic methadone and levomethadone have not been examined in opioid-dependent patients.

To address these issues, we performed a region-wide cross-sectional study in opioid-dependent patients in MMT in Berlin, Germany. To study potential tolerance development and differences between methadone and levomethadone on tolerance development, we collected data on dosage and duration of MMT in patients receiving either methadone or levomethadone.

Firstly, we hypothesized that long-term methadone treatment in opioid-dependent patients results in a tolerance development. Secondly, we hypothesized that treatment with racemic methadone compared to levomethadone leads to a reduced tolerance development.

## Methods

The study was approved by the local ethics of the Charité, Universitätsmedizin Berlin. In our survey, 720 patients participated who were treated with racemic methadone or levomethadone and gave information on the dosage of treatment. Out of these, 679 patients indicated the duration of MMT.

The survey was performed from May to October 2011. All 20 hospitals and 110 practices in Berlin licensed to offer MMT were approached in order to reach patients under MMT fulfilling the DSM IV criteria of opioid dependence. Ten psychiatric hospitals and 47 practices participated in the study, 29 practices reported to have the license, but had stopped offering MMT, and 34 practices and 10 hospitals declined participation. The participating hospitals and practices were located in 10 out of the 12 districts of Berlin. In Berlin, 5032 patients were registered to receive substances of opioid maintenance treatment.

Patients were informed about the questionnaire both orally and in writing. The questionnaire was completed anonymously, without the help of staff, and collected in a sealed box.

### Measurement

Data were collected on substance of treatment, age, sex, education, duration of MMT (treatment duration without interruptions), duration of dependence, continued use of illegal drugs despite MMT, dosage, imprisonment, withdrawal treatment in the past, comorbid psychiatric disorders, and chronic infection. Additionally, the questionnaire involved questions on side effects, frequency of outpatient treatment, and valuation of maintenance treatment. Different types of questions were used: grouping questions were used to collect age and duration of opioid dependence in order to maintain professional secrecy. Dosage of substitute and duration of MMT and school education were asked using open questions. All other items contained single or multiple-choice questions. The questionnaire was designed for this study.

Survey data concerning side effects, take-home treatment, and valuation of treatment have been previously published [28–31]. Anonymity of data was part of the ethical agreement.

### Statistical analysis

Data was analyzed using PASW Statistics 20 employing chi-square and *t* test. Correlation analyses were performed by using partial correlations with the covariate sex. Comparison of correlation was performed via the Fisher *z*-transformation.

## Results

A total of 679 patients reported the type of substance, dosage, and duration of MMT. Out of these, 370 patients (54.5 %) reported treatment with racemic methadone and 309 (45.5 %) with levomethadone. The mean dosage of methadone (mean dosage of racemic methadone and levomethadone in methadone dose equivalent) was 94.1 mg per day (standard deviation (SD) 43.8). Mean dosage of methadone of patients treated for 1 year or less (number of patients (N) 51) was 81.6 mg per day (SD 38.3), and patients treated for more than 20 years (N 26) reported a dosage of 125.4 mg (SD 42.3). This is an increase of dosage by a factor of 1.5.

Patients recruited in hospital or practices did not differ concerning mean dosage of methadone dose equivalent (hospital 89.9 mg, SD 38.1; practices 94.5 mg, SD 44.8; *p* = 0.406, *T* = 0.8).

The mean duration of MMT of the 679 included patients was 7.5 years (SD 5.9), with a minimum duration of 1 month and a maximum duration of 30 years.

Out of the 679 patients, 628 were 1 year or longer in MMT. Out of these, 339 patients (54.0 %) reported treatment with racemic methadone and 289 (46.0 %) with levomethadone.

### Clinical data

To characterize patients treated with racemic methadone in comparison to those receiving levomethadone, these two groups were compared with regard to age, sex, years of education, years of MMT, years of dependency, continued use of illegal substances, continued use of multiple illegal substances, mean dosage of methadone dose equivalent, imprisonment, withdrawal treatment in the past, comorbid psychiatric disorders, and chronic infection (see Table 1). These analyses revealed that patients treated with racemic methadone were significantly more often male (75.3 %) compared to patients treated with levomethadone (65.7 %) (*p* = 0.006). Patients treated with levomethadone were significantly longer in MMT (8.3 years) compared to patients with racemic methadone (6.8 years) (*p* = 0.002) and also received significantly higher dosages of methadone equivalent (99.1 mg) compared to patients with racemic methadone (90.0 mg) (*p* = 0.007). We did not find statistically significant differences between both groups for other factors (see Table 1).

**Table 1:** Clinical data

Clinical data^a^	Substance		Statistic
	Methadone	Levomethadone	*p* value
	(*N* = 370)	(*N* = 309)	
Age (years)			
18–20	6 (1.6)	1 (0.3)	0.474^c^; *Χ* = 0.54
21–30	84 (22.7)	65 (21.0)	
31–40	102 (27.6)	87 (28.2)	
41–50	135 (36.5)	117 (37.9)	
51–60	35 (9.5)	37 (12.0)	
61–70	8 (2.2)	2 (0.6)	
Sex			
Male	275 (75.3)	203 (65.7)	0.006; *Χ* = 7.5
Female	90 (24.7)	106 (34.3)	
Years of education mean ± SD	10.3 ± 1.7	10.4 ± 1.4	0.751; *T* = −.32
Years of methadone maintenance treatment mean ± SD	6.8 ± 5.6	8.3 ± 6.2	0.002; *T* = −3.2
Years of dependency			
≤1	1 (0.3)	3 (1.1)	1.0^b^; *Χ* = 0.007
≥1–3	9 (2.7)	9 (3.2)	
≥3–5	25 (7.6)	15 (5.3)	
≥5–10	68 (20.6)	39 (13.8)	
≥10	227 (68.8)	216 (76.6)	
Continued use of illegal drugs despite MMT	226 (63.8)	205 (67.9)	0.285; *Χ* = 1.2
Continued use of multiple illegal drug despite MMT	82 (23.6)	81 (27.6)	0.275; *Χ* = 1.3
Methadone dose/methadone equivalent (mg)^b^ mean ± SD	90.0 ± 41.3	99.1 ± 46.9	0.007; *T* = −2.7
Imprisonment in the past	212 (60.7)	176 (59.1)	0.688; *Χ* = 1.9
Withdrawal treatment in the past	172 (50.0)	159 (53.5)	0.372; *X* = 0.8
Comorbid psychiatric disorder^e^	90 (26.4)	99 (33.3)	0.06; *X* = 3.7
Chronic infection	180 (52.2)	144 (48.2)	0.31; *X* = 1.0
HIV	18 (5.2)	25 (8.3)	0.117; *X* = 2.5
Hepatits C	163 (47.2)	124 (41.5)	0.141; *X* = 2.1
Hepatitis B	25 (7.2)	27 (9.0)	0.407; *X* = 0.6
Lues	1 (0.3)	2 (0.7)	0.483; *X* = 0.5

^a^Data shown as *N* (%) if not otherwise specified. Out of the 679 patients who specified type of substance, dosage, and duration of MMT, some patients did not gave information concerning further data: 5 concerning gender, 67 concerning duration of dependence, 31 concerning detoxification therapies, 23 concerning continued use of illegal drugs, 42 concerning continued use of multiple illegal drugs, and 32 concerning imprisonment

^b^Dosage of levomethadone was calculated into equivalent methadone dosage

^c^Chi-square for group ≤30 versus >30 age (years)

^d^Chi-square for group ≤5 versus >5 years of dependency

^e^Multiple answers were possible. Comorbid psychiatric disorders reported *N* > 5: depression *N* = 108; psychotic disorder and schizophrenia *N* = 13; anxiety disorders *N* = 30; personality disorder *N* = 39; and ADHD *N* = 10

### Correlation analyses

In a first step, dose equivalents were considered conjointly for racemic methadone and levomethadone. This analysis revealed a significant correlation between dosage and duration of treatment (*p* = 0.000008; *R* = 0.171). Specifically, higher dosages were found in patients indicating a longer duration of MMT. In a second step, each substance was examined separately. This analysis revealed a significant correlation between dosage of racemic methadone and duration of treatment (*p* = 0.001; *R* = 0.171) as well as between dosage of levomethadone and duration of treatment (*p* = 0.006; *R* = 0.158). This correlation remained significant, when only patients receiving MMT for 1 year or longer were considered (*p* = 0.00008, *R* = 0.157 for conjoint analysis; *p* = 0.007, *R* = 0.148 for racemic methadone; *p* = 0.007, R = 0.157 for levomethadone).

### Fisher *z*-transformation

Correlations between dosage and duration of treatment were compared between racemic methadone and levomethadone using the Fisher *z*-transformation. This comparison revealed no significant difference (*p* = 0.86; *z* = 0.17). Moreover, no significant difference between substances was found when only patients receiving MMT for 1 year or longer were considered (*p* = 0.91; *z* = 0.1).

## Discussion

The data of our study show a significant correlation between duration of treatment and dosage for both substances racemic methadone and levomethadone. Longer duration of treatment was associated with higher dosages, which can be interpreted as a tolerance development. This correlation remained significant when only patients receiving MMT for 1 year or longer were considered. Thus, even after early adjustment of dosage in the first year, there is a gradual increase of dosage over time, which can be interpreted as a long-term tolerance development.

In our data, we found a 1.5-fold increase of dosage over 20 years of MMT. The majority of participants in our study were long-term MMT patients, some with up to 30 years of treatment. Our data may be of clinical relevance especially for patients in long-term treatment. That is, one could assume that the gradual loss of efficacy of methadone may require a successive increase of dosage to maintain the beneficial effects of MMT. Our results on long-term treatment add to other studies of short treatment intervals, showing that tolerance development of methadone seems to be reduced compared with other opioids such as morphine [18, 23].

Our data show significant correlations between duration of treatment and dosage, for both substances racemic methadone and levomethadone. These correlations did not differ significantly between these substances, indicating that tolerance development is not dependent on the type of substance. Although we can only speculate on this finding, it may be that the NMDA antagonism has only minor impact on the long-term development of tolerance, or is less relevant compared to other mechanisms influencing tolerance, such as the internalization of the μ-receptor.

Although the current study comprised more than 600 patients, it may be that the sample was too small to detect a difference in tolerance development between two substances.

Our data is based on a cross-sectional analysis, which allowed us the inclusion of patients with up to 30 years of MMT. However, the cross-sectional design also comes with a few limitations: Firstly, it cannot be ruled out that dosing practice has changed over the past 20 to 30 years. Patients who have been in MMT for a long time might have received higher initial dosages than commonly applied these days. On the other hand, just in the past 10 years, guidelines, for example of the Cochrane database, tended to recommend higher dosages to prevent relapse [32, 33]. Hence, one would rather expect higher dosages in the past years, which would result in a negative correlation, which we did not find. Additionally, if it was for this bias, it would be equally for both substances. Secondly, racemic methadone and levomethadone differed significantly in duration of treatment and mean dosage. On the other hand, comparison of tolerance (expressing changes of mean dosage over time) focuses on the difference in gradient, which is not affected by the height and length of the gradient. Thirdly, the study is based on self-reported data, which might limit the quality of results concerning concomitant use of illegal substances or comorbid psychiatric disorders. On the other hand, the survey was performed anonymously, and there was no reason of not reporting, e.g., concomitant use of illegal substances. Additionally, it is very likely that patients answered correctly concerning the data we focused on: type of substance, dosage, and duration of treatment. Fourthly, we cannot exclude patients misusing substances of MMT over time, which might drive increasing dosage. On the other hand, this misuse might represent the need of higher dosage in terms of tolerance. However, we cannot exclude that single persons received higher dosages without taking medication, but selling it instead [34].

Nevertheless, long-term randomized prospective trials are needed to examine the development of methadone tolerance. Such trials are difficult to carry out due to high relapse rates and the precondition of blindness of dosage, which few opioid users would accept.

The mean dosages in our sample were between 90 and 100 mg methadone dose equivalent per day. These dosages are in accordance with the recommended dosages of a review of the Cochrane Collaboration, showing higher effectiveness in higher dosages [33]. Patients in our sample had mean treatment duration of 7.5 years, and some patients were up to 30 years in MMT. These long periods of treatment stand both for the effectiveness of MMT and for a growing number of patients permanently remaining in MMT programs [30, 35–38].

## Conclusions

Our data show an increase of dosage in patients with methadone and levomethadone, which could be interpreted as tolerance development under long-term MMT. However, tolerance development did not differ significantly between racemic methadone and levomethadone. Our study has some limitations, such as the cross-sectional design and the self-reported data. Nonetheless, our data may be relevant for long-term MMT patients. When patients and their staff physicians observe a decrease of opioid efficacy during MMT (e.g., relapse, or symptoms of craving), tolerance development could be a contributing factor.

## References

[1] Ward J, Hall W, Mattick RP (1999). Role of maintenance treatment in opioid dependence. Lancet.

[2] Holland R, Maskrey V, Swift L, Notley C, Robinson A, Nagar J et al. Treatment retention, drug use and social functioning outcomes in those receiving 3 months versus 1 month of supervised opioid maintenance treatment. Results from the Super C randomized controlled trial. Addiction. 2013. doi:10.1111/add.12439. 10.1111/add.12439 24304349

[3] Wang PW, Lin HC, Yen CN, Yeh YC, Hsu CY, Chung KS (2015). Comparison of outcomes after 3-month methadone maintenance treatment between heroin users with and without HIV infection: a 3-month follow-up study. Harm Reduction J.

[4] Wang K, Fu H, Longfield K, Modi S, Mundy G, Firestone R (2014). Do community-based strategies reduce HIV risk among people who inject drugs in China? A quasi-experimental study in Yunnan and Guangxi provinces. Harm Reduction J.

[5] Ball JC, Lange WR, Myers CP, Friedman SR (1988). Reducing the risk of AIDS through methadone maintenance treatment. J Health Soc Behav.

[6] Ward J, Darke S, Hall W, Mattick R (1992). Methadone maintenance and the human immunodeficiency virus: current issues in treatment and research. Br J Addict.

[7] Caplehorn JR, Dalton MS, Haldar F, Petrenas AM, Nisbet JG (1996). Methadone maintenance and addicts’ risk of fatal heroin overdose. Subst Use Misuse.

[8] Wilson P, Watson R, Ralston GE (1994). Methadone maintenance in general practice: patients, workload, and outcomes. BMJ.

[9] Kwan TH, Wong NS, Lee SS (2015). Participation dynamics of a cohort of drug users in a low-threshold methadone treatment programme. Harm Reduction J.

[10] Coid J, Carvell A, Kittler Z, Healey A, Henderson J. Opiates, criminal behaviour, and methadone treatment. 2000.

[11] Gossop M, Mardsen J, Stewart D (2001). NTORS after five years: the National Treatment Outcome Research Study; changes in substance use, health and criminal behaviour during the five years after intake.

[12] Havinga P, van der Velden C, de Gee A, van der Poel A (2014). Differences in sociodemographic, drug use and health characteristics between never, former and current injecting, problematic hard-drug users in the Netherlands. Harm Reduction J.

[13] Dole VP, Nyswander M (1965). A medical treatment for diacetylmorphine (heroin) addiction: a clinical trial with methadone hydrochloride. JAMA.

[14] Dole VP, Nyswander ME, Kreek MJ (1966). Narcotic blockade—a medical technique for stopping heroin use by addicts. Trans Assoc Am Phys.

[15] Dole VP, Joseph H (1978). Long-term outcome of patients treated with methadone maintenance. Ann N Y Acad Sci.

[16] Dole VP, Joseph H (1977). Methadone maintenance: outcome after termination. N Y State J Med.

[17] Inturrisi CE (2005). Pharmacology of methadone and its isomers. Minerva Anestesiol.

[18] Trafton JA, Ramani A (2009). Methadone: a new old drug with promises and pitfalls. Curr Pain Headache Rep.

[19] Corkery JM, Schifano F, Ghodse AH, Oyefeso A (2004). The effects of methadone and its role in fatalities. Hum Psychopharmacol.

[20] Freye E, Latasch L (2003). Development of opioid tolerance—molecular mechanisms and clinical consequences. AINSAnasthesiol Intensivmed Notfallmed Schmerzther.

[21] Koch T, Hollt V (2008). Role of receptor internalization in opioid tolerance and dependence. Pharmacol Ther.

[22] Williams JT, Ingram SL, Henderson G, Chavkin C, von Zastrow M, Schulz S (2013). Regulation of mu-opioid receptors: desensitization, phosphorylation, internalization, and tolerance. Pharmacol Rev.

[23] Arttamangkul S, Quillinan N, Low MJ, von Zastrow M, Pintar J, Williams JT (2008). Differential activation and trafficking of micro-opioid receptors in brain slices. Mol Pharmacol.

[24] Silverman SM (2009). Opioid induced hyperalgesia: clinical implications for the pain practitioner. Pain Physician.

[25] Morgan MM, Christie MJ (2011). Analysis of opioid efficacy, tolerance, addiction and dependence from cell culture to human. Br J Pharmacol.

[26] Davis AM, Inturrisi CE (1999). d-Methadone blocks morphine tolerance and N-methyl-D-aspartate-induced hyperalgesia. J Pharmacol Exp Ther.

[27] Holtman JR, Wala EP (2007). Characterization of the antinociceptive and pronociceptive effects of methadone in rats. Anesthesiology.

[28] Bald LK, Bermpohl F, Heinz A, Gallinat J, Gutwinski S (2013). Heroin or conventional opioid maintenance? The patients’ perspective. J Addict Med.

[29] Gutwinski S, Bald LK, Heinz A, Muller CA, Schmidt AK, Wiers C (2013). Take home maintenance medication in opiate dependence. Deutsches Arzteblatt Int.

[30] Gutwinski S, Bald LK, Gallinat J, Heinz A, Bermpohl F (2013). Why do patients stay in opioid maintenance treatment?. Subst Use Misuse.

[31] Schoofs N, Riemer T, Bald LK, Heinz A, Gallinat J, Bermpohl F et al. Methadone and levomethadone—dosage and side effects.. Psychiatrische Praxis. 2013. doi:10.1055/s-0033-1349627. 10.1055/s-0033-1349627 24254424

[32] Bakker A, Fazey C (2006). Methadone tolerance testing in drug misusers. BMJ.

[33] Faggiano F, Vigna-Taglianti F, Versino E, Lemma P (2003). Methadone maintenance at different dosages for opioid dependence. Cochrane database Syst Rev.

[34] Iwersen-Bergmann S, Jungen H, Andresen-Streichert H, Muller A, Elakkary S, Puschel K (2014). Intravenous methadone application as a serious risk factor for an overdose death: methadone-related fatalities in Hamburg from 2007 to 2012. Int J Legal Med.

[35] Kimber J, Copeland L, Hickman M, Macleod J, McKenzie J, De Angelis D (2010). Survival and cessation in injecting drug users: prospective observational study of outcomes and effect of opiate substitution treatment. BMJ.

[36] Amato L, Davoli M, Perucci CA, Ferri M, Faggiano F, Mattick RP (2005). An overview of systematic reviews of the effectiveness of opiate maintenance therapies: available evidence to inform clinical practice and research. J Subst Abus Treat.

[37] Milby JB (1988). Methadone maintenance to abstinence. How many make it?. J Nerv Ment Dis.

[38] Magura S, Rosenblum A (2001). Leaving methadone treatment: lessons learned, lessons forgotten, lessons ignored. Mt Sinai J Med.

